# Factors affecting spine–femur discordance in the percentage of young adult mean on dual-energy X-ray absorptiometry in the elderly population: a retrospective study

**DOI:** 10.1186/s12891-022-05015-3

**Published:** 2022-01-21

**Authors:** Shoshi Akiyama, Takaaki Tanaka, Jun Udaka, Naoya Inagaki, Yoshio Kumagae, Masaaki Chazono, Tatsuki Matsuoka, Mitsuru Saito

**Affiliations:** 1 Department of Orthopaedic Surgery, NHO Utsunomiya National Hospital, 2160 Shimo-Okamoto, Utsunomiya, Tochigi 329-1193 Japan; 2 Department of Orthopaedic Surgery, Jikei University School of Medicine, 3-25-8 Nishi-Shimbashi, Minato-ku Tokyo, 105-8461 Japan

**Keywords:** Spine–femur discordance, Young adult mean, Dual-energy X-ray absorptiometry, Osteoporosis, Lumbar spine, Bone mineral density

## Abstract

**Background:**

Several retrospective studies have reported spine–femur discordance in bone mineral density (BMD) values. However, the average age of individuals in these studies was the mid-50s, which is younger than the typical age of individuals requiring treatment for primary osteoporosis. Therefore, we aimed to investigate factors associated with discordance in the percentage of young adult mean (YAM) between the lumbar spine and femoral neck in the elderly population.

**Methods:**

We evaluated 4549 dual-energy X-ray absorptiometry (DXA) measurements obtained from 2161 patients (269 men and 1892 women) between January 2014 and December 2017 at our hospital. For individuals with more than one eligible set of measurements, the first record was used. We investigated each patient’s age, sex, body mass index, current smoking status, alcohol consumption, use of steroids, presence of diabetes mellitus, and presence of rheumatoid arthritis.

**Results:**

The mean age of the patients was 76.4 ± 8.9 years. Older age (*p* <  0.001), male sex (*p* <  0.001), and diabetes mellitus (*p* = 0.007) were significantly associated with spine–femur discordance in the percentage of YAM.

**Conclusion:**

The frequency and magnitude of spine–femur discordance in the percentage of YAM from DXA scans increased with age. Notably, more than 77.4% of patients in their 90s had spine–femur discordance > 10% of YAM. Furthermore, the frequency of spine–femur discordance was higher in men and in patients with diabetes mellitus, suggesting that the percentage of YAM at the lumbar spine may not be reliable for diagnosis of osteoporosis in patients with these factors.

## Background

Dual-energy X-ray absorptiometry (DXA) is a cost-effective method for measuring bone mineral density (BMD) with low radiation exposure [1]. Strategies for initiating treatment in patients with primary osteoporosis frequently rely on the use of DXA. Investigating whether fragility fractures are present, measuring BMD, and considering the presence of other diseases suggestive of secondary osteoporosis are crucial in the management of osteoporosis [2]. In Japan, the diagnostic criteria for osteoporosis defined in 1996 were initially based on the percentage of young adult mean (YAM) for the areal BMD of the lumbar spine (LS) or femoral neck (FN), which are as follows: normal, more than 80% of YAM; osteopenia, less than or equal to 80% and more than 70% of YAM; and osteoporosis, presence of a fragility fracture or less than or equal to 70% of YAM. Further, in 2012, the classification was revised to include T-scores, which are as follows: normal, more than or equal to 1.0 T-score; osteopenia, less than − 1.0 and more than − 2.5 T-score; and osteoporosis, presence of a proximal femur or vertebral body fracture or presence of other fragility fractures with less than 80% of YAM or less than or equal to 70% of YAM [3]. Further, 70 and 80% of YAM correspond to T-scores of − 2.5 and almost − 1.7, respectively [3]. Therefore, a difference of 10% in the percentage of YAM is significant in terms of diagnosing osteoporosis.

In contrast, BMD is measured at a single site in many countries. FN BMD was reported to have the highest predictive value for all major osteoporotic fractures, especially hip fractures [2]. However, patients with low LS and FN BMD have different types of fractures. It is therefore advisable to measure BMD at multiple sites in order to select the appropriate treatment [4]. We often find in clinical practice that patients have spine–femur discordance in BMD values assessed using the percentage of YAM or T-score [5]. In such patients, there is a risk that necessary treatment for osteoporosis will not be administered depending on which site is measured. Spine–femur discordance in BMD values occur for physiological, pathological, anatomical, artifactual, and technical reasons [6]. Several retrospective cohort studies have reported that age, obesity, menopause, and multiple pregnancies are associated with the development of spine–femur discordance in BMD [7–9]. However, the average age of individuals in those studies was the mid-50s, which is younger than the typical age of individuals who require treatment for primary osteoporosis.

In Japan, a country with the world’s largest elderly population, the number of older adults requiring nursing care continues to increase, and locomotive syndrome, including osteoporosis, affects a large proportion (25%) of the elderly population [10]. However, no studies have yet analyzed discordance in the percentage of YAM in the elderly population. Therefore, this study aimed to investigate the actual incidence and prevalence of spine–femur discordance and factors associated with discordance in the percentage of YAM, predominantly in the elderly Japanese population.

## Methods

### Patient population

This was a retrospective study of DXA data obtained from January 2014 to December 2017 at our hospital. Data from 4549 DXA scans of the LS and FN acquired simultaneously in a group of patients aged > 50 years were evaluated. For individuals with more than one eligible set of measurements, the first record was used. All patients underwent general interviews and physical measurements of height and weight before the DXA scans. Details on current smoking status, high alcohol consumption (more than three drinks a day), presence of diabetes mellitus, use of corticosteroids, and rheumatoid arthritis were obtained for each patient. The study was conducted in accordance with The Code of Ethics of the World Medical Association (Declaration of Helsinki) and was approved by the institutional review board of our hospital. The requirement for informed consent was waived owing to the retrospective nature of this study.

### DXA measurement method

All DXA scans were acquired either for diagnosing osteoporosis or for determining the efficacy of medication using a Hologic Discovery Ci system (Hologic Inc., Marlborough, MA).

The measurement accuracy (coefficient of variation) of the DXA method was 1.0% for both the LS and FN. LS BMD values were measured at the L2 to L4 level in the anteroposterior view. DXA scans of the FN were obtained with the patient in the supine position and lower limbs internally rotated by 20°. BMD values obtained for the right FN were primarily used; however, values obtained from the left FN were used when the right hip was replaced with implants. The exclusion criteria were patients with implants in the LS and both hips. The Japanese normative data for the percentages of YAM at LS and FN reported by the Japanese Society of Bone and Mineral Research and the Joint Review Committee of the Japanese Society for Osteoporosis were based on the average values for people aged 20–44 and 20–29 years, respectively. In the present study, LS (L2–4) BMD was calculated using a reference YAM of 1.024 g/cm^2^ (SD = 0.131 g/cm^2^) for men and 1.010 g/cm^2^ (SD = 0.119 g/cm^2^) for women. FN BMD was calculated using a reference YAM of 0.828 g/cm^2^ (SD = 0.092 g/cm^2^) for men and 0.790 g/cm^2^ (SD = 0.090 g/cm^2^) for women [3].

### Statistical analysis

Discordance in the percentage of YAM (percentage of YAM at LS minus percentage of YAM at FN) was categorized according to the following thresholds: more than or equal to 10% (group A); more than or equal to − 10% to less than 10% (group B); and less than − 10% (group C). Continuous variables are expressed as means ± standard deviations and were analyzed using analysis of variance and Student’s t-test. Categorical variables are expressed as numbers (percentages) and were compared using Fisher’s exact test. A Bonferroni post hoc test was performed for multiple comparisons. All tests were two-tailed, and statistical significance was defined as *p* <  0.05. All statistical analyses were performed using SPSS version 23.0 (IBM Japan, Ltd., Tokyo, Japan).

## Results

The patient demographics are presented in Table 1. The study population included 2161 patients (269 men [12.4%] and 1892 women [87.6%]) with a mean age of 76.4 ± 8.9 years. The mean age of male and female patients was 76.2 ± 9.2 and 76.4 ± 8.8 years, respectively. The numbers of patients in their 50s, 60s, 70s, 80s, and 90s were 74, 446, 736, 799, and 106, respectively. The number of male patients in their 50s, 60s, 70s, 80s, and 90s was 10, 57, 93, 93, and 16, respectively, while the corresponding number of female patients was 64, 389, 643, 706, and 90, respectively. There were 32 patients (1.5%) with a body mass index > 30 kg/m^2^, 85 (3.9%) who currently smoked, 60 (2.8%) who had high alcohol consumption 242 (11.2%) with diabetes mellitus, 270 (12.5%) who were using corticosteroids, and 174 (8.1%) with rheumatoid arthritis.

**Table 1:** Characteristics of the study population (*n* = 2161)

	All	Age group				
	Mean age: 76.4 ± 8.9(years)	50s	60s	70s	80s	90s
*n* (%)	2161 (100%)	74 (3.4%)	446 (20.6%)	736 (34.1%)	799 (37.0%)	106 (4.9%)
Sex						
Male, *n* (%)	269 (12.4%)	10	57	93	93	16
Female, *n* (%)	1892 (87.6%)	64	389	643	706	90
Body mass index > 30 kg/cm^2^, *n* (%)	32 (1.5%)	4	8	12	7	1
Current smoking, *n* (%)	85 (3.9%)	4	33	40	6	2
High alcohol comsumption, *n* (%)	60 (2.8%)	3	17	28	10	2
Diabetes mellitus, *n* (%)	242 (11.2%)	13	48	87	92	2
Corticosteroid use, *n* (%)	270 (12.5%)	17	66	73	107	7
Rheumatoid arthritis, *n* (%)	174 (8.1%)	6	58	52	55	3
Mean percentage of YAM at LS (%)	79.1 ± 18.5	82.5 ± 20.8	80.8 ± 18.9	80.4 ± 18.3	77.5 ± 18.0	72.5 ± 17.9
Mean percentage of YAM at FN (%)	67.5 ± 14.4	75.1 ± 13.5	73.7 ± 14.1	69.2 ± 13.0	63.3 ± 13.8	55.3 ± 13.6

Data are presented as mean ± standard deviation or *n* (%).

*LS* lumbar spine, *FN* femoral neck, *YAM* young adult mean

The mean percentages of YAM in the LS among patients (total, male, and female) in their 50s, 60s, 70s, 80s, and 90s were: 82.5% ± 20.8, 83.4% ± 20.9, and 82.3% ± 21.0%; 80.8% ± 18.9, 89.2% ± 21.6, and 79.6% ± 18.2%; 80.4% ± 18.3, 91.5% ± 22.8, and 78.8% ± 17.0%; 77.5% ± 18.0, 86.4% ± 20.5, and 76.3% ± 17.3%; and 72.5% ± 17.9, 79.0% ± 11.8, and 71.3% ± 18.6%, respectively. The corresponding mean percentages of YAM in the FN were 75.1% ± 13.5, 75.8% ± 17.5, and 75.0% ± 12.9%; 73.7% ± 14.1, 77.9% ± 17.1, and 73.1% ± 13.5%; 69.2% ± 13.0, 75.2% ± 15.4, and 68.3% ± 12.4%; 63.3% ± 13.8, 67.5% ± 16.6, and 62.8% ± 13.3%; and 55.3% ± 13.6, 60.1% ± 7.4, and 54.4% ± 14.3%, respectively.

Patient demographics in the three groups based on spine–femur discordance in the percentages of YAM are shown in Table 2. Older age (*p* <  0.001), male sex (p <  0.001), and diabetes mellitus (*p* = 0.007) were significantly associated with spine–femur discordance in the percentage of YAM. There was a significant difference in age among the groups, with patients in group A being significantly older than those in groups B and C (78.0 ± 8.7 years vs. 74.8 ± 8.7 years and 73.2 ± 8.5 years, *p* <  0.001). The proportion of male patients was significantly higher in group A than in group B (15.4% vs. 9.0%, p <  0.001). The proportion of patients with diabetes mellitus was also significantly higher in group A than in group B (13.3% vs. 8.9%, *p* = 0.007). No significant difference in any of the factors investigated was found between groups B and C. Body mass index > 30 kg/m^2^, current smoking, high alcohol consumption, corticosteroid use, and rheumatoid arthritis were not associated with spine–femur discordance in the percentage of YAM.

**Table 2:** Comparison among the groups of discordance in the percentage of YAM (*n* = 2161)

	Group A: ≥ 10 (*n* = 1129, 52.2%)	Group B: ≥ −10 to < 10 (*n* = 887, 41.0%)	Group C: < −10 (*n* = 145, 6.7%)	*p*-value			
				for all	A vs. B	A vs. C	B vs. C
Age (years)	78.0 ± 8.7	74.8 ± 8.7	73.2 ± 8.5	< 0.001^a^	< 0.001^b^	< 0.001^b^	0.106^b^
Male, *n* (%)	174 (15.4%)	80 (9.0%)	15 (10.3%)	< 0.001^c^	< 0.001^d^	0.406^d^	> 0.999^d^
BMI > 30 kg/cm^2^, *n* (%)	20 (1.8%)	11 (1.2%)	16 (0.7%)	0.528^c^			
Current smoking, *n* (%)	38 (3.4%)	37 (4.2%)	10 (6.9%)	0.111^c^			
High alcohol comsumption, *n* (%)	35 (3.1%)	23 (2.6%)	2 (1.4%)	0.542^c^			
Diabetes mellitus, *n* (%)	150 (13.3%)	79 (8.9%)	13 (9.0%)	0.006^c^	0.007^d^	0.556^d^	> 0.999^d^
Corticosteroid use, *n* (%)	156 (13.8%)	99 (11.2%)	15 (10.3%)	0.154^c^			
Rheumatoid arthritis, *n* (%)	100 (8.9%)	64 (7.2%)	17 (6.9%)	0.376^c^			
Percentage of YAM at LS (%)	87.9 ± 18.3	70.5 ± 13.3	63.1 ± 10.4	< 0.001^a^	< 0.001^b^	< 0.001^b^	< 0.001^b^
Percentage of YAM at FN (%)	64.3 ± 14.6	69.6 ± 13.1	79.3 ± 12.0	< 0.001^a^	< 0.001^b^	< 0.001^b^	< 0.001^b^

Data are presented as mean ± standard deviation or *n* (%).

*BMI* Body mass index, *LS* lumbar spine, *FN* femoral neck, *YAM* young adult mean

*p*-value: ^a^ ANOVA; ^b^ unpaired t test with Bonferroni correction; ^c^ Fisher’s exact test; ^d^ Fisher’s exact test with Bonferroni correction

**Fig. 1:**
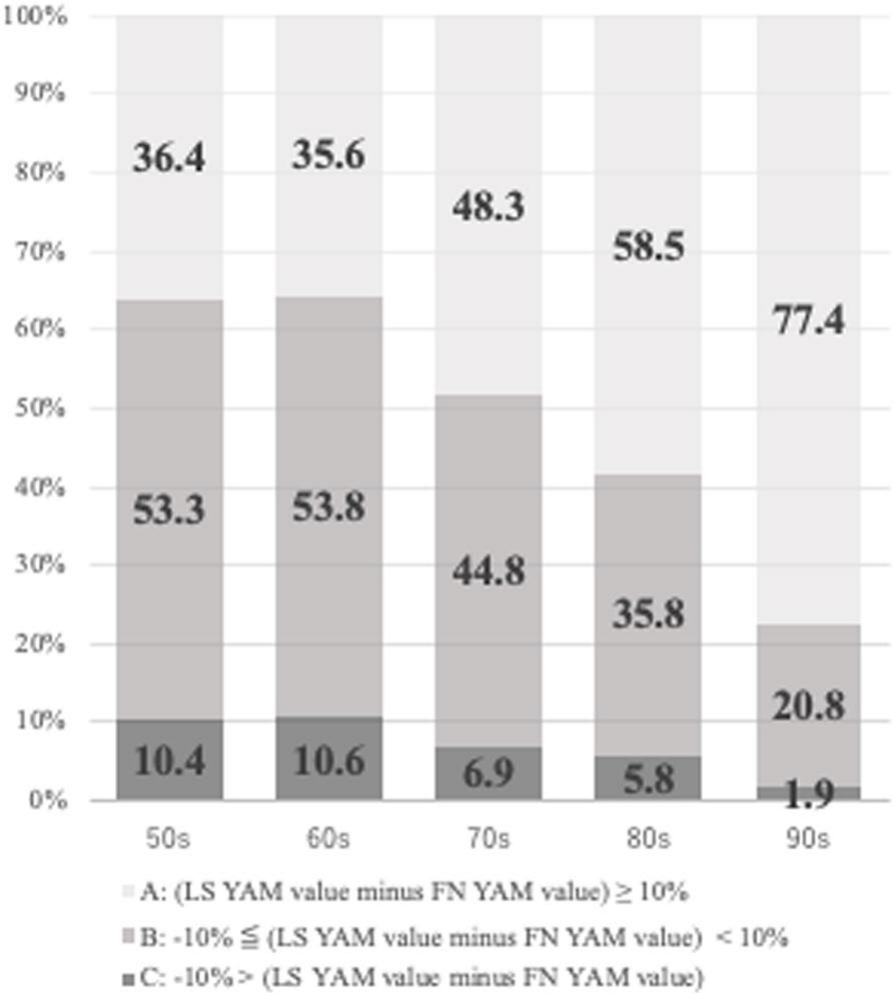
Discordance in the percentage of YAM (percentage of YAM at LS minus percentage of YAM at FN) was categorized according to the following thresholds: <− 10% (group A); ≥ − 10 to < 10% (group B); and ≥ 10% (group C). YAM, young adult mean; LS, lumbar spine; FN, femoral neck

The distribution of spine–femur discordance by age group is presented in Fig. 1. The discordance rates in groups A, B, and C were respectively 36.4, 53.2, and 10.4% for patient in their 50s, 35.6, 53.8, and 10.6% for those in their 60s, 48.3, 44.8, and 6.9% for those in their 70s, 58.4, 35.8, and 5.8% for those in their 80s, and 77.4, 20.7, and 1.9% for those in their 90s.

**Fig. 2:**
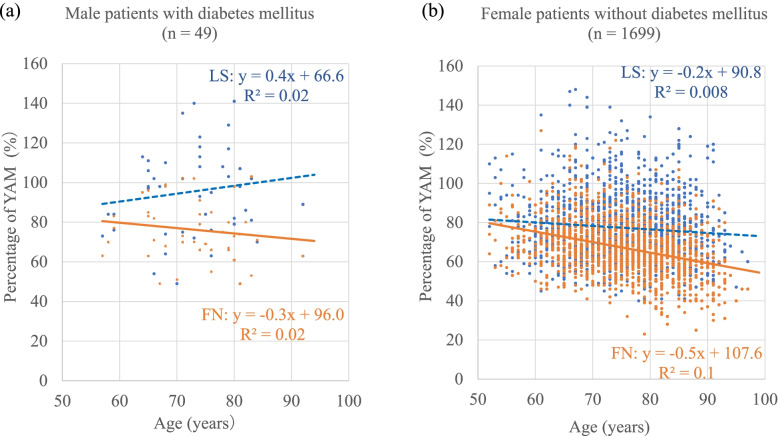
The percentage of YAM at LS and FN according to age. Each dot represents one person. The dashed and solid lines approximate the percentage of YAM at LS and FN, respectively. YAM, young adult mean; LS, lumbar spine; FN, femoral neck

Figure 2 shows the relationship between older age and the percentages of YAM at LS and FN in male patients with diabetes mellitus and in female patients without diabetes mellitus. The regression equations and correlation coefficients (R^2^) were as follows: (a) male patients with diabetes mellitus: the percentage of YAM at LS, y = 0.4x + 66.6 (R^2^ = 0.02); the percentage of YAM at FN, y = − 0.3x + 96.0 (R^2^ = 0.02) and (b) female patients without diabetes mellitus: the percentage of YAM at LS, y = − 0.2x + 90.8 (R^2^ = 0.008); the percentage of YAM at FN, y = − 0.5x + 107.6 (R^2^ = 0.1).

### One example of discordance

**Fig. 3:**
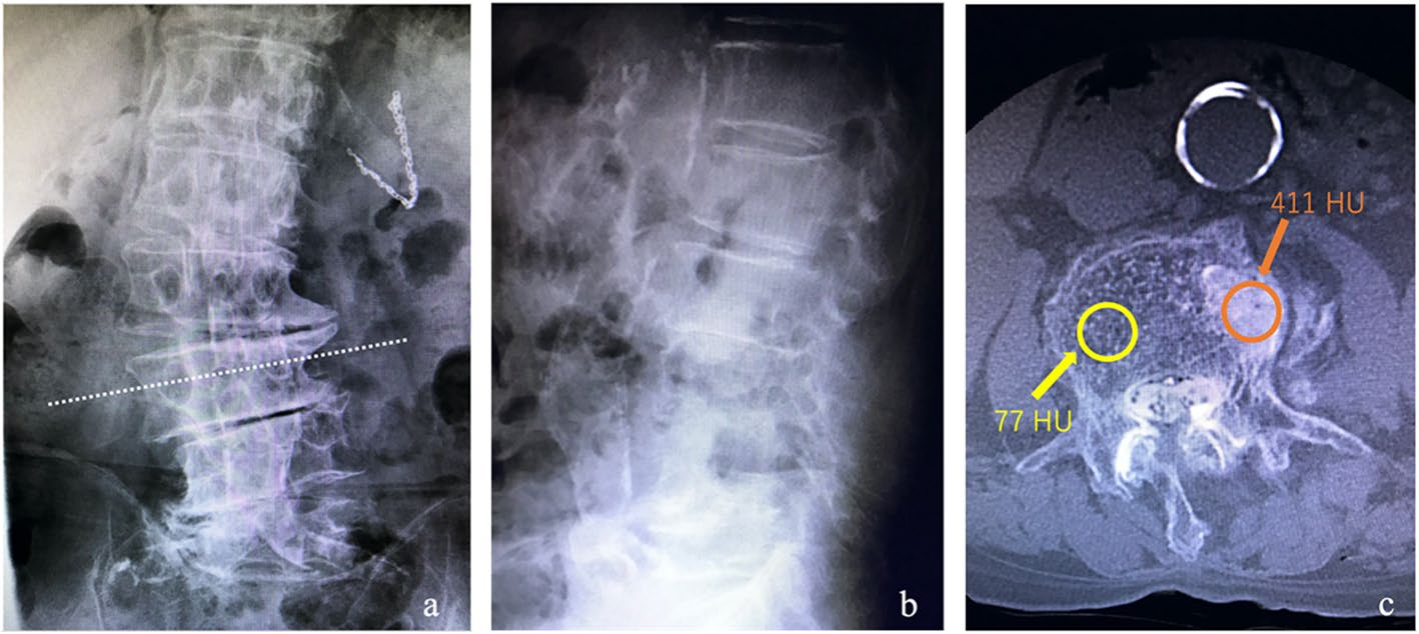
**a** An anteroposterior radiograph. **b** A lateral radiograph. **c** An axial computed tomography image of the fourth lumbar vertebra reconstructed at the level of the dotted line in the anteroposterior radiograph (a)

A 79-year-old man with type 2 diabetes mellitus was found to have degeneration of the LS. There was a large discordance in BMD on DXA between the percentages of YAM at LS and FN (93 and 67%, respectively). Osteophytes and aortic calcification were identified on radiographs (Fig. 3a, b), which increased the LS YAM values. The axial computed tomography (CT) values for the fourth lumbar vertebra were 411 Hounsfield units and 77 Hounsfield units with and without osteosclerosis, respectively (Fig. 3c).

## Discussion

To the best of our knowledge, this is the first study to examine the factors associated with discordance in the percentage of YAM in a population with an average age of > 75 years. In fact, we found two new findings. One is that male is a significant factor in spine–femur discordance in the percentage of YAM. Another one is that percentage of YAM at lumbar spine in the elderly men with diabetes mellitus increased with age. These two findings have not been reported in the previous mid-50s analyses.

Using multivariate logistic regression analyses, Mounach et al. showed that age, menopause, and obesity contributed to discordant spine–femur T-scores in 3479 patients with a mean age of 55.7 years [7]. Moayyeri et al. investigated 4299 patients with an average age of 53.4 years and reported the causes of discordant T-scores to be age, obesity, menopause, and late menopause [8]. Furthermore, Singh et al. reported a significant association of discordant T-scores with age > 50 years, premature menopause, and multiple pregnancies [9]. The average age of the individuals in the aforementioned studies was in the mid-50s, which is younger than the typical age of patients requiring treatment for primary osteoporosis [3].

Our study population closely reflects the age range of patients with osteoporosis in today’s aging society. Similar to previous reports, this study found age to be the most important factor associated with spine–femur discordance in the percentage of YAM. Notably, 58.5% of patients in their 80s and 77.4% of those in their 90s had a spine–femur discordance rate of > 10% of YAM (group A). Age-related pathological findings, such as osteophytes, facet hyperplasia, endplate sclerosis, and aortic calcification, are more prominent in the LS than in the FN [ 11–14]. In contrast, FN BMD is affected by osteoarthritis of the hip joint [15]. Yoshimura et al. reported that the prevalence of advanced-stage hip arthrosis was 3.5% (men, 1.0%; women, 6.1%), which is much lower than that of degenerative lumbar spondylosis [16].

Our findings show that sex is also a significant factor in spine–femur discordance in the percentage of YAM, which is a novel finding. This study focused on the elderly and its findings may be epidemiologically plausible based on data from the Research on Osteoarthritis/Osteoporosis Against Disability study, which showed that the prevalence of deformity associated with lumbar spondylosis with a Kellgren–Lawrence grade ≥ 2 was 85.3 and 74.8%, respectively, for men and women in their 70s and 89.6 and 78.3% for men and women aged > 80 years [12]. Moreover, Mitchell et al. reported that abdominal aortic calcification was more severe in men than in women in 336 cases at autopsy [17]. Szulc P reported that clinical risk factors for abdominal aortic calcification include genetic factors, males, heavy smokers, high fat diet, mainly abdominal obesity, type 2 diabetes mellitus, metabolic syndrome, hypertension, and dyslipidemia [18]. He also reported that severe abdominal aortic calcification is associated with lower volumetric BMD assessed by central QCT (lumbar spine, hip, femoral neck), faster bone loss and higher risk of major fragility fracture [18]. However, DXA has a high absorption range with abdominal aortic calcification as an artifact. Accordingly, the prevalence of lumbar degeneration and abdominal aortic calcification in men is higher than that in women, which may lead to an increase in LS BMD on DXA.

This study also showed that diabetes mellitus was a significant independent predictor of increased spine–femur discordance in the percentage of YAM. Interestingly, the percentage of YAM at LS for men with diabetes mellitus increased with age (Fig. 2a). This is a rare finding and was not observed for any other factors. Although the correlation coefficient was not particularly high, an in-depth investigation of the reason behind this is necessary. In contrast, when male sex and diabetes mellitus were excluded, women without diabetes mellitus showed much less spine–femur discordance in the percentage of YAM at the corresponding age than did men with diabetes mellitus (Fig. 2a, b). Hong et al. recently showed that the prevalence of diabetes mellitus was higher in both men and women when the LS T-score was higher than the FN T-score [19]. However, the reason was not clarified. A meta-analysis indicated that type 2 diabetes mellitus affected both LS and FN BMD [20]. Increased BMD has been reported in patients with type 2 diabetes due to obesity and abnormal adipokine secretion [21]. Special management strategies are needed for patients with type 2 diabetes because their bones are fragile; they have porous cortical bone due to increased levels of advanced glycation end products [22]. The reasons for the increase in spine–femur discordance in the percentage of YAM in patients with diabetes mellitus are presumably as follows. Diffuse idiopathic skeletal hyperostosis (DISH), a condition characterized by ossification of the anterior longitudinal ligament of the spine, generally increases the BMD of the spine [23]. Although the etiological factors have not yet been elucidated, HLA-B8 is common in both DISH and diabetes [24]. At an average age of 65 years, 10.8% of Japanese individuals (22.0% male, 2.5% female) had DISH [25]. Moreover, aortic calcification, which is common in diabetic patients, contributed to increased LS BMD [26], and may be a cause of the spine–femur discordance in the percentage of YAM. Calcification of the femoral artery had no effect because it was not anatomically within the measurement field of the FN on DXA. In contrast, the thicker abdominal aorta had more impact as it was in the measurement field of the LS.

CT bone mineral densitometry expressed as Hounsfield units is not yet routinely used, is not widely available. We do not recommend CT scans for diagnosis of osteoporosis. However, CT scans obtained previously for the work-up for visceral diseases may be useful for vertebral Hounsfield units measurements in the elderly population given that elderly people often undergo CT scans due to other illnesses. Recently, measuring the CT value of lumbar cancellous bone in the area while excluding degenerative changes and vascular calcifications has proven useful for evaluating BMD [27]. The Hounsfield units measurement had excellent inter-rater and intra-rater reliability. Pickhardt et al. reported that Hounsfield units values of 110 and 135, respectively, were 90% specific for detecting osteoporosis and osteopenia at L1 [28]. Abdominal CT scans previously performed for visceral disease could be useful for vertebral Hounsfield units measurement in the elderly population. Thoracic vertebral CT values have also been reported to be useful for detecting bone loss [29]. Therefore, CT assessment may be an alternative method for measuring local BMD in cases with spine–femur discordance in the percentage of YAM due to degenerative change and aortic calcification. A limitation of CT is the consequent radiation exposure, which is low for one DXA scan of the LS (1.7 μGy) [30], but substantially higher for a CT scan at the same site (10 mGy) [31].

This study has several limitations that should be acknowledged. First, there were fewer men than women in the population studied, which would be expected given that osteoporosis is a more prevalent disease in women. However, there were 269 men in the study, which was considered sufficient for statistical analysis. Second, we included degenerative vertebrae and vertebral fractures, whose prevalence increases with age, were included in the study. Therefore, a nested case-control study is necessary to investigate factors other than aging in depth. Third, we excluded patients who had implants in both hips because the FN BMD was unmeasurable. Many patients with hip fractures were expected to have low FN BMD. Contrastingly, most cases of total hip replacement were done due to hip osteoarthritis that would not cause low FN BMD. Finally, the study population included patients who had undergone DXA testing for diagnosis of osteoporosis as well as those who were being treated with drugs for osteoporosis. Most drugs used to prevent or treat osteoporosis lead to formation of more cancellous bone than cortical bone. The LS, which is rich in cancellous bone, tended to have a higher increase in the percentage of YAM than the FN, owing to the administration of bisphosphonates and parathyroid hormone products [32]. Therefore, the first test value was used to minimize the effects of medication in patients undergoing multiple DXA tests.

## Conclusion

Our results indicate that spine–femur discordance in the percentage of YAM tends to be greater in patients who were older, those who are male, and those with diabetes mellitus. Therefore, the percentage of YAM at LS may be inappropriate for diagnosing osteoporosis in patients with these factors.

## Data Availability

The datasets generated during the current study are available from the corresponding author on reasonable request.

## Abbreviations

BMD: Bone mineral density
CT: Computed tomography
DISH: Diffuse idiopathic skeletal hyperostosis
DXA: Dual-energy X-ray absorptiometry
FN: Femoral neck
LS: Lumbar spine
YAM: Young adult mean

## References

[1] Jain RK, Vokes T (2017). Dual-energy X-ray absorptiometry. J Clin Densitom.

[2] Lewiecki EM, Watts NB, McClung MR, Petak SM, Bachrach LK, Shepherd JA (2004). Official positions of the international society for clinical densitometry. J Clin Endocrinol Metab.

[3] Soen S, Fukunaga M, Sugimoto T, Sone T, Fujiwara S, Endo N (2013). Diagnostic criteria for primary osteoporosis: year 2012 revision. J Bone Miner Metab.

[4] Alarkawi D, Bliuc D, Nguyen TV, Eisman JA, Center JR (2016). Contribution of lumbar spine BMD to fracture risk in individuals with T-score discordance. J Bone Miner Res.

[5] Nelson DA, Molloy R, Kleerekoper M (1998). Prevalence of osteoporosis in women referred for bone density testing: utility of multiple skeletal sites. J Clin Densitom.

[6] Woodson G (2000). Dual X-ray absorptiometry T-score concordance and discordance between the hip and spine measurement sites. J Clin Densitom.

[7] Mounach A, Abayi DA, Ghazi M, Ghozlani I, Nouijai A, Achemlal L (2009). Discordance between hip and spine bone mineral density measurement using DXA: prevalence and risk factors. Semin Arthritis Rheum.

[8] Moayyeri A, Soltani A, Tabari NK, Sadatsafavi M, Hossein-Neghad A, Larijani B (2005). Discordance in diagnosis of osteoporosis using spine and hip bone densitometry. BMC Endocr Disord.

[9] Singh M, Magon N, Singh T (2012). Major and minor discordance in the diagnosis of postmenopausal osteoporosis among Indian women using hip and spine dual-energy X-ray absorptiometry. J Midlife Health.

[10] Ishibashi H (2018). Locomotive syndrome in Japan. Osteoporos Sarcopenia.

[11] Pappou IP, Girardi FP, Sandhu HS, Parvataneni HK, Cammisa FP, Schneider R (1976). Discordantly high spinal bone mineral density values in patients with adult lumbar scoliosis. Spine (Phila Pa).

[12] Muraki S, Yamamoto S, Ishibashi H, Horiuchi T, Hosoi T, Orimo H (2004). Impact of degenerative spinal diseases on bone mineral density of the lumbar spine in elderly women. Osteoporos Int.

[13] Rand T, Seidl G, Kainberger F, Resch A, Hittmair K, Schneider B (1997). Impact of spinal degenerative changes on the evaluation of bone mineral density with dual energy X-ray absorptiometry (DXA). Calcif Tissue Int.

[14] Li S, Yin L, Li K, Hu B, Wang L, Wang Y (2020). Relationship of volumetric bone mineral density by quantitative computed tomography with abdominal aortic calcification. Bone.

[15] Preidler KW, White LS, Tashkin J, McDaniel CO, Brossmann J, Andresen R (1997). Dual-energy X-ray absorptiometric densitometry in osteoarthritis of the hip. Influence of secondary bone remodeling of the femoral neck. Acta Radiol.

[16] Yoshimura N, Campbell L, Hashimoto T, Kinoshita H, Okayasu T, Wilman C (1998). Acetabular dysplasia and hip osteoarthritis in Britain and Japan. Br J Rheumatol.

[17] Mitchell JR, Adams JH (1977). Aortic size and aortic calcification: a necropsy study. Atherosclerosis.

[18] Szulc P (2016). Abdominal aortic calcification: a reappraisal of epidemiological and pathophysiological data. Bone.

[19] Hong AR, Kim JH, Lee JH, Kim SW, Shin CS (2019). Metabolic characteristics of subjects with spine-femur bone mineral density discordances: the Korean National Health and Nutrition Examination Survey (KNHANES 2008–2011). J Bone Miner Metab.

[20] Ma L, Oei L, Jiang L, Estrada K, Chen H, Wang Z (2012). Association between bone mineral density and type 2 diabetes mellitus: a meta-analysis of observational studies. Eur J Epidemiol.

[21] Ducy P, Amling M, Takeda S, Priemel M, Schilling AF, Beil FT (2000). Leptin inhibits bone formation through a hypothalamic relay: a central control of bone mass. Cell.

[22] Saito M, Marumo K (2010). Collagen cross-links as a determinant of bone quality: a possible explanation for bone fragility in aging, osteoporosis, and diabetes mellitus. Osteoporos Int.

[23] Sohn S, Chung CK, Han I, Park SB, Kim H (2018). Increased bone mineral density in cervical or thoracic diffuse idiopathic skeletal hyperostosis (DISH): a case-control study. J Clin Densitom.

[24] Belanger TA, Rowe DE (2001). Diffuse idiopathic skeletal hyperostosis: musculoskeletal manifestations. J Am Acad Orthop Surg.

[25] Kagotani R, Yoshida M, Muraki S, Oka H, Hashizume H, Yamada H (2015). Prevalence of diffuse idiopathic skeletal hyperostosis (DISH) of the whole spine and its association with lumbar spondylosis and knee osteoarthritis: the ROAD study. J Bone Miner Metab.

[26] Bendix EF, Johansen E, Ringgaard T, Wolder M, Starup-Linde J (2018). Diabetes and abdominal aortic calcification-a systematic review. Curr Osteoporos Rep.

[27] Choi MK, Kim SM, Lim JK (2016). Diagnostic efficacy of Hounsfield units in spine CT for the assessment of real bone mineral density of degenerative spine: correlation study between T-scores determined by DEXA scan and hounsfield units from CT. Acta Neurochir (Wien).

[28] Pickhardt PJ, Pooler BD, Lauder T, del Rio AM, Bruce RJ, Binkley N (2013). Opportunistic screening for osteoporosis using abdominal computed tomography scans obtained for other indications. Ann Intern Med.

[29] Mao SS, Li D, Syed YS, Gao Y, Luo Y, Flores F (2017). Thoracic quantitative computed tomography (QCT) can sensitively monitor bone mineral metabolism: comparison of thoracic QCT vs lumbar QCT and dual-energy X-ray absorptiometry in detection of age-relative change in bone mineral density. Acad Radiol.

[30] Damilakis J, Perisinakis K, Vrahoriti H, Kontakis G, Varveris H, Gourtsoyiannis N (2002). Embryo/fetus radiation dose and risk from dual X-ray absorptiometry examinations. Osteoporos Int.

[31] Tremblay E, Thérasse E, Thomassin-Naggara I, Trop I (2012). Quality initiatives: guidelines for use of medical imaging during pregnancy and lactation. Radiographics.

[32] McClung MR, San Martin J, Miller PD, Civitelli R, Bandeira F, Omizo M (2005). Opposite bone remodeling effects of teriparatide and alendronate in increasing bone mass. Arch Intern Med.

